# Assessment of Potential Health Risks Associated with the Intake of Heavy Metals in Fish Harvested from the Largest Estuary in Colombia

**DOI:** 10.3390/ijerph17082921

**Published:** 2020-04-23

**Authors:** Carlos H. Pinzón-Bedoya, Martha L. Pinzón-Bedoya, José Pinedo-Hernández, Iván Urango-Cardenas, José Marrugo-Negrete

**Affiliations:** 1 Departamento de Química, Laboratorio de Toxicología y gestión ambiental, Facultad de Ciencias Básicas, Universidad de Córdoba, Carrera 6 No. 76-103 Montería 230002, Córdoba, Colombia; cpinzon63@hotmail.com (C.H.P.-B.); josejph@hotmail.com (J.P.-H.); ivanurango@correo.unicordoba.edu.co (I.U.-C.); 2 Programa de Ingeniería Ambiental, Universidad de Pamplona, 543057 Pamplona, Colombia; mlpinzon@unipamplona.edu.co

**Keywords:** trace metals, fish, potential risk, human health, Colombia

## Abstract

This study assesses the potential human health risks posed by six heavy metals (Hg, As, Pb, Cd, Cu, and Zn) found in five of the most consumed fish species (*Mugil incilis, Centropomus undecimalis, Cathorops mapale, Eugerres plumieri,* and *Elops smithi*) collected by the riverine population living in Ciénaga Grande de Santa Marta (CGSM), the largest estuary in Colombia. Metal concentrations were low compared with those reported in other regions around the world and the maximum value established by international monitoring organizations. The estimation of the potential risk (HQ) indicated that Cu and Hg could generate negative effects in groups of women of childbearing age (WCA) and the remaining population (RP), because they exceeded their related reference doses, with HQ values > 1; however, Cu and Hg concentrations were not high in fish and EWI, MFW, or MeHgPSL values shows that there is no evidence of a potential health risk from MeHg exposure in the study population. Therefore, the recommendations are to establish continuous monitoring of heavy metals together with strategies that address the high fish consumption, as well as to implement mechanisms for the mitigation of contamination of the watershed, to ensure the safety of organisms in the ecosystem and human health, not only of populations who depend on aquatic resources in the area but also of those that market and consume these resources in the Colombian Caribbean.

## 1. Introduction

Contamination with heavy metals in aquatic ecosystems is considered a serious problem at the global level due to the effects on ecosystems and their importance for human health. Heavy metals such as copper (Cu) and zinc (Zn) play a vital role in the development of some specific metabolic functions of biological systems and are considered essential because of their requirement in small concentrations in living organisms [1]; however, their deficiency or excess can lead to health problems [2]. Several authors agree in warning—from an environmental perspective—that it is necessary to be concerned about the characteristics of some heavy metals, particularly regarding their non-degradability and bioaccumulation through food chains [3,4]. Among the heavy metals that demand attention, cadmium (Cd), lead (Pb), mercury (Hg), and arsenic (As) are highlighted. These are considered non-essential and with toxic effects on the ecosystem and for humans, depending on their concentration, chemical nature, or oxidation state [5].

Ciénaga Grande de Santa Marta (CGSM) is the largest marsh complex in Colombia, directly influenced by rivers from the western slopes of Sierra Nevada de Santa Marta (SNSM) and the Magdalena River, which make it a strategic aquatic ecosystem [6]. However, in this important area of the country, concentrations of some heavy metals have already been reported in water, sediments, plant communities, and various organisms [7,8,9]. Furthermore, the values found have, in some cases, exceed the permissible limits set by the United States Environmental Protection Agency (USEPA), the Codex Alimentarius, and the Joint FAO/World Health Organization (WHO) Expert Committee on Food Additives (JECFA). Previous reports of heavy metals in different environmental matrices show the existence of risks to human health and the ecosystem, due to the bioaccumulation and biomagnification processes that can occur in the food chain. For example, fish demand particular attention since their intake constitutes one or perhaps the only source of protein of the population, being a vital resource in the area of influence of this lagoon body; moreover, it is also one of the most important commercialization lines of the region [4]. Accordingly, the aim of this study was to determine the concentrations of essential (Cu, Zn) and toxic (As, Cd, Hg, Pb) heavy metals in muscle tissue in five (5) fish species of high consumption and commercial interest: *Mugil incilis* (common, grey or Parassi mulet (English); lisa rayada (Spanish)), *Centropomus undecimalis* (common snook (English); róbalo blanco (Spanish)), *Cathorops mapale* (mapale sea catfish (English); chivo mapalé (Spanish)), *Eugerres plumieri* (striped mojarra (English); mojarra rayada (Spanish)), and *Elops smithi* (malacho (English); macabí (Spanish)), to assess their accumulation and the risk to human health through their consumption.

## 2. Materials and Methods

### 2.1. Study Area

**Figure 1:**
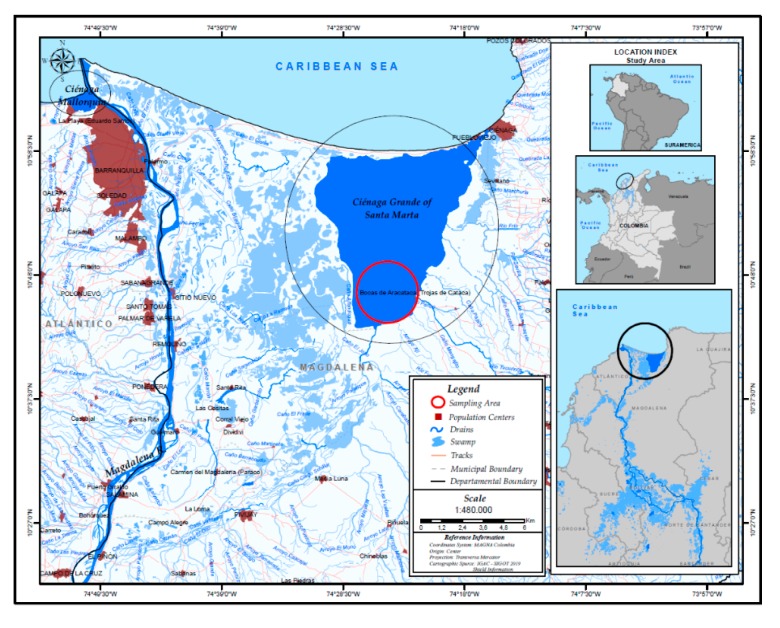
Map of Ciénaga Grande de Santa Marta in Colombia, outlining the study area.

Ciénaga Grande de Santa Marta (CGSM), Santa Marta-Colombia, is the largest estuarine coastal lagoon in Colombia (1321 km^2^), located in the north of the country on the Caribbean coast, more specifically in the department of Magdalena (10°20′–11°05′ N and 74°06′–74°52′ W) (Figure 1). It is permanently connected to the Caribbean Sea through the mouth of La Barra, and indirectly to the Magdalena River through natural and artificial channels. The estuary is surrounded by mangroves (*Rhizophora* spp.), and its waters have an average annual depth of 1.5 m and a temperature of 30 °C [10,11]. It is a highly populated area (eight settlements) with high socio-economic interest; for decades, its inhabitants have depended directly on artisanal fisheries.

### 2.2. Fish Sampling and Chemical Analysis

Fish were collected during the fishing campaigns with local fishermen between January and December 2018. The species sampled included those considered as the most important in the diet of the population of the area (Table 1). The length and weight of the fish were measured; then, the samples were individually packaged in labeled in polyethylene bags, refrigerated at 4 °C and transported to the laboratory. Subsequently, fish samples were identified using the FishBase database (http://www.fishbase.org) and the information provided by fishermen. Following the procedure described by UNEP/IOC/IAEA/FAO, the pectoral fin of the left side next to the skin was removed, and with a knife, a portion of 3 cm wide was cut out [12].

The following analytical method used to determine the total Hg concentration in tissue samples was applied. A fish sample of 0.02 g (wet weight, ww) was collected and then introduced into the direct mercury analyzer DMA 80 Tricell Milestone, according to the EPA method 7473 [13]. For Cu, Zn, Cd, and Pb analyses, the AOAC Official Method 999.11 of dry ashing was followed for the extraction of fish muscle samples [14]. For the analysis of As, 1 g of sample mixed with Mg(NO_3_)_2_ at 550 °C in a muffle furnace was calcined. Subsequently, 1 mL of concentrated HNO_3_ was added and heated to dryness, and finally, it was redissolved with 4.5 N HCl, filtered through a 0.45 μm filter, and refilled up to a volume of 25 mL with distilled water [15]. Analyses were performed using a Thermo Scientific iCE™ 3500 AAS Atomic Absorption Spectrometer coupled to a VP100 Continuous Flow Vapor Generator (Waltham, MA, USA) (As) (HGAAS; [16]) and a GFS35Z Integrated Zeeman Graphite Furnace (Waltham, MA, USA) ((Cd, Pb, Cu, Zn) (GFAAS; [17]). The analytical quality control of the method was evaluated in triplicate with the certified reference materials (CRM) IAEA 407 and DORM-4. The different concentrations of metals established with the certified values and the recovery percentage ranged between 92%–96%. The detection limits for the different metals were 0.006 µg/g for Cd, 0.010 µg/g for Pb, 0.05 µg/g for Cu, 0.016 µg/g for Zn, 0.014 µg/g for Hg, and 0.016 µg/g for As.

### 2.3. Human Health Risk Assessment

The study to estimate the potential human health risk was based on data from a survey on fish consumption in different locations in CGSM, as well as on calculations of the estimated dietary intake (EDI), hazard quotient (HQ), maximum allowable fish consumption rate (CRlim), and metal pollution index (MPI), as previously described in other studies [18,19].

In the current study, a dietary survey was conducted by interviewing 215 residents settled around CGSM, indicating that on average, the frequency of fish consumption was six times or more per week, and the species commonly consumed and marketed according to the survey were: *Euguerres plumieri*, followed by *Mugil incilis, Elop smithi, Cathorops mapale*, and *Centropomus undecimalis*. The persons surveyed were classified into three age groups: children (CHD: 1–15 years old), women of childbearing age (WCA: 16–48 years old), and the remaining population (RP: men > 15 years old and women ≥ 49 years old).

Additionally, in this study, total arsenic (inorganic + organic) concentration was quantified in fish samples. Several studies have shown that in fish, this metalloid is found mainly in the form of arsenobetaine, a non-toxic organic form, and the rest is inorganic arsenic, which is highly toxic and carcinogenic [20]. However, fish samples were not analyzed for inorganic arsenic, therefore, for all risk assessment methods (EDI, HQ, and CRlim), 10% of total arsenic was assumed to be inorganic arsenic, according to the worst-case scenario established by the USEPA for health risk assessment of As intake due to fish consumption [21].

#### 2.3.1. Estimated Daily Intake

The estimated daily intake (EDI) (µg/kg body weight/day) was calculated using the following equation:

(1) $$EDI=\frac{C_{m}\cdot DI}{BW}$$

where *C_m_* is the mean heavy metal concentration in the fish muscle tissue (µg/g), *DI* is the fish intake consumed per day (g/day), and *BW* is the mean body weight of the person (kg). In this study, body weight averages of 37 kg for CHD, 69 kg for WCA, and 73 kg for RP were registered from the survey, as well as a daily fish intake of 50 g for CHD, 279 g for WCA, and 243 g for RP. The reference doses (RfD, µg/kg BW/day, defined as the maximum tolerable daily intake of a specific metal that does not result in any deleterious health effects) employed for the heavy metals studied were those established by the JECFA [22], the United States Environmental Protection Agency (EPA, Washington, DC, USA) [23], and the Agency for Toxic Substances and Disease Registry (ATSDR, Atlanta, GA, USA) [24] (Table 2).

#### 2.3.2. Hazard Quotient

The hazard quotient (HQ), defined as the relationship between the EDI of a heavy metal in relation to its reference dose, was used to characterize the potential risk.

(2) $$HQ=\frac{EDI}{RfD\;}$$

There is no risk if HQ < 1; however, if HQ > 1, then there is a potential risk associated with the heavy metal considered.

#### 2.3.3. Maximum Allowable Fish Consumption

The Equation (3) below is used to calculate the maximum allowable fish consumption rate (CRlim, in g/day) of contaminated fish with a non-carcinogenic effect [23].

(3) $$CR_{lim}=\frac{RfD\cdot BW}{C_{m}}$$

#### 2.3.4. Risk Associated with Methylmercury

The potential risk of human exposure to Hg was assessed with the estimated weekly intake of MeHg (EWI_MeHg_) per kg of body weight of the studied individual (µg/kg BW/week) using the equation described by UNEP (Geneva, Switzerland) [25]:

(4) $$EWI_{MeHg}=\frac{WFC\cdot C_{MeHg}}{BW}$$

where *WFC* is the weekly intake (g/week) of fish; *C_MeHg_* is the median concentration of MeHg (µg/kg) in fish calculated considering that most of the Hg in fish is MeHg (higher than 80%), and most of the MeHg ingested through fish consumption is quickly absorbed into the body [26,27]; *BW* is the body weight of the person (kg).

Additionally, the permissible safety level (MeHg_PSL_), which is the concentration of MeHg that the consumed fish species should contain to avoid exceeding the provisional tolerable weekly intake (PTWI) of MeHg established by JECFA (1.6 µg/kg BW/week), was calculated using the following equation [26]:

(5) $${\left[MeHg\right]}_{PSL}=\frac{C_{m}\cdot PTWI}{WI_{MeHg}}$$

Likewise, the maximum estimated amount of fish (in g) that people should eat weekly to avoid Hg exposure (MFW) was calculated using the following equation:

(6) $$MFW=\frac{PTWI\cdot WFC}{WI_{MeHg}}$$

### 2.4. Metal Pollution Index

The metal pollution index (MPI) was evaluated to compare the total content of metals in different fish species. This index was obtained by calculating the geometrical mean of the metal levels analyzed in fish [19,28].

(7) MPI (µg/g)=(Cf1 x Cf2 x … Cfn) 1/n

where *Cfn* is the concentration of a metal in the sample.

### 2.5. Statistical Analysis

The results of the heavy metal concentrations for each fish species are presented as the mean ± standard deviation of the samples analyzed. An exploratory analysis using the Kolmogorov–Smirnov test to demonstrate the normality of the data showed a normal distribution. Differences between fish species were evaluated using a Student’s-test. Pearson’s correlation analysis was used to establish the relationships between the variables. Statistical analyses were performed with the SPSS 10.5 software, establishing a confidence level of 95%.

## 3. Results

### 3.1. Heavy Metals Concentrations in Fish

Table 1 shows the different species of fish collected that are most commonly marketed and the heavy metal concentrations found in CGSM. The species with the highest frequency were carnivores, including *Cathorops mapale, Centropomus undecimalis*, and *Elops smithi*, which represent 60% of the collected samples. On the other hand, the other 40% corresponds to the species *Eugerres plumieri* (euryphagous diet) and *Mugil incilis* (detritivorous features). Conversely, in descending order according to local fish consumption by the population in the area *Euguerres plumieri* (92%) is the most consumed species, followed by *Mugil incilis* (77%), *Elop smithi* (64%), *Cathorops mapale* (54%), and *Centropomus undecimalis* (35%). Individuals of the *Cathorops mapale* species showed the lowest total length and weight averages. *Elops smithi* showed the highest average total length, and *Centropomus undecimalis* had the highest average weight.

Except for Hg (r = 0.60; *p* < 0.05), the length and weight did not show a statistically significant correlation with the concentration of the heavy metals assessed. As a function of average heavy metal concentrations, in descending order we found Cu > Zn > As > Pb > Hg > Cd. The concentration of heavy metals by species and according to the feeding habit does not show statistically significant differences (*p* > 0.05). *Elops shmiti* is the only species that follows a food web pattern of carnivores > non-carnivores, showing the highest concentrations of Cu, Hg, and Pb. This is different from what was found in the species *Eugerres plumieri* that recorded the highest levels of Zn. On the other hand, the species *Mugil incilis* showed higher levels of Cd and As.

### 3.2. Health Risk Assessment

The EDI results for the different population groups were found to be between 0.02–16.8 for CHD, 0.04–40.5 for WCA, and 0.04–37.4 for RP (Table 2). The EDI in descending order according to the population group was RP > WCA > CHD. Cd contributed with the lowest daily intake, while Cu and Zn contributed with the highest daily intake; the values obtained were between 600 to 900 times higher than the lowest EDI value.

**Figure 2:**
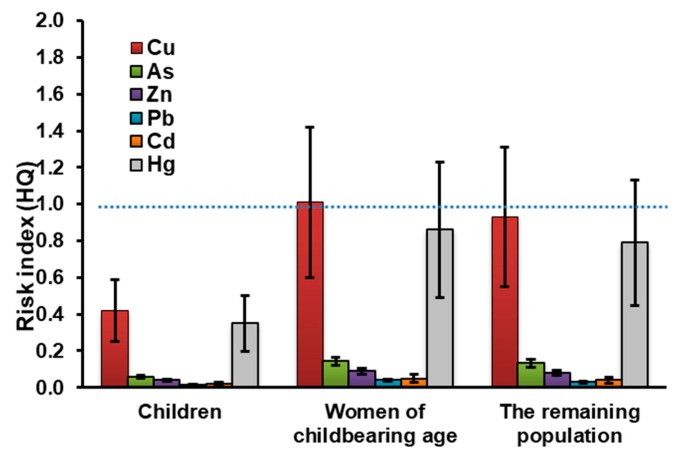
Hazard quotient (HQ) for the heavy metals assessed in fish species for different population groups.

On the other hand, reports of the maximum allowable fish consumption rate (CRlim) are higher for all evaluated age groups and close to the average daily fish consumption for the groups of WCA (279 g/day) and RP (243 g/day) in the case of Cu (Table 2). Similarly, the HQ shows potential risk by Cu for these two population groups by presenting values of HQ > 1 or very close to HQ = 1; WCA (Cu: HQ = 1.01), and RP (Cu: HQ = 0.93) (Figure 2).

When considering the metal pollution index (MPI) per species in descending accumulation order, we find *Elops smithi* > *Mugil incilis* > *Centropomus undecimalis* > *Cathorops mapale* > *Eugerres plumieri* (Table 1).

The main exposure route of MeHg is through fish consumption [40]; therefore, the potential risk in the population was assessed based on the estimated weekly intake (EWI), the maximum amount of fish that can be consumed weekly per person (MFW) without adverse health effects, and the permissible safety level of MeHg (MeHg_PSL_) in fish for human consumption (Table 2). The EWI values found were below the provisional tolerable weekly intake values for MeHg of 1.6 μg/kg BW/week (WCA and CHD) and 3.2 μg/kg BW/week (RP). The amount of fish consumed weekly according to the population survey was between 350 and 1953 g, which is less than the results obtained for the MFW estimate. The MeHg_PSL_ was far superior compared to what was obtained through the laboratory analysis of the harvested fish.

## 4. Discussion

Hg concentration showed a statistically significant correlation with the length and weight of the fish, evidencing a mercury bioaccumulation process in the aquatic biota in the ecosystem, as reported by Marrugo et al. for fish species in the large marshes of Achí and Ayapel in the Mojana region of Colombia [41]. In all species, the concentrations of Zn and Cu were found to be the highest, explaining possibly why these metals play a fundamental role in the enzymatic and respiratory processes of fish [42]. Moreover, the average concentration of heavy metals does not exceed the values established by FAO/WHO [37], the European Union [38], and the Colombian maximum limits for heavy metals in fish [39]. Hg, Pb, and Cu were the only metals to follow a food web pattern of carnivores > non-carnivores. In the case of Hg, it is known that it accumulates in the aquatic food chain, with higher concentrations in predatory fish [18,41,43,44]. For other metals, the concentration is mostly controlled by the habitat, eating habits, metal accumulation capacity, and the type of organism [42,45].

Nonetheless, it is well known that *Elops smithi* feeds on a combination of fish and crabs (http://www.fishbase.org), which results in higher bioaccumulation of Cu, Hg, and Pb compared to the specie *Eugerres plumieri* that has a lower trophic level. However, high levels of Zn in this species may be associated with feeding on micro-bivalves and detritus found in the sediments. This is dissimilar to the diet of *Mugil incilis* comprised of phytoplankton, zooplankton, and debris, where the concentration of Cd and As may be associated with transfer factors in the aquatic environment. In addition, species-specific characteristics in some fishes that present this type of food habitat may result in the variation of the concentration of essential or toxic metals [46]. In general, heavy metals have been determined in marine organisms other than fish in CGSM such as those found 30 years ago by Campos [47], where high concentrations (µg/g) of Cd (2–11), Pb (0.86–6), and Zn (200–950) were found in oysters; these results indicated a significant variation of the concentration depending on the sampling points for the ecosystem [47]. Therefore, studies of the content and bioavailability of heavy metals should be carried out according to spatial distribution in sediments as well as in different organisms. This should be done to establish translocation and bioaccumulation factors, so contamination areas and anthropogenic sources of contamination can be considered. When compared with other studies, we observe that the Pb, Zn, and Cd values were lower than the ones registered in other studies (Table 1), except for those reported by Fernández-Maestre et al. [9] on the Colombian Caribbean coast. Hg concentrations were slightly lower than those reported in the Mallorquín swamp [18] and also in the Atrato River in Colombia [36]. The concentrations of As were found to be much lower than those reported by Li et al. [35] in the Spratly Islands in China, but similar to the ones found by Gallego et al. [36] in the Atrato River Delta in Colombia. However, Cu concentrations were higher compared to the results reported locally and internationally. Of the metals assessed in fish, the lowest concentration was found for Cd, coinciding with different reports indicated in Table 1 and other studies in marine organisms of the Mediterranean and Black Seas [35,48].

When comparing the content by species at a local level in ecosystems with similar geographical characteristics or for the same study area, the Hg concentrations for the species *Mugil incilis* and *Eugerres plumieri* were similar to a study reported for CGSM [7]. Besides, the concentrations of Cd, Pb, Hg, and Zn were slightly lower in the species *Eugerres plumieri* and *Centropomus undecimalis* in contrast to the Cu concentrations that showed higher values, compared to those reported for the Mallorquín swamp located in the Colombian Caribbean area [18].

Fuentes et al. [18] reported similar results regarding the contribution of each metal in the EDI value but differed in their order according to the population group, as follows: CHD > WCA > RP for the consumption of fish species collected in the Mallorquín swamp in Colombia [18]. Cu in the WCA and RP groups pose a risk to their health because the calculated EDI results are above the tolerable intake reference levels established by the JECFA, the USEPA, and the ATSDR (Cu: 40 μg/kg/day). On the other hand, the HQ shows potential risk by Cu for WCA and RP (Figure 2). Although Cu is considered an essential micronutrient for humans, high levels of this metal easily lead to Fenton-type redox reactions, which could, in turn, lead to oxidative damage and cell death [49]. In the case of As, to avoid overestimating the health risk of As intake via the consumption of fish, an assumption about the percent of inorganic As in fish (10%) had to be made [21,50]; HQ results (<1) for As suggested that non-carcinogenic health effects from the intake of arsenic in the fish species are not expected for consumers.

HQ for Zn indicates no potential risk, although concentration levels are higher than the other analyzed metals. It is also well known that Zn is considered in the literature as an essential element, and its dietary excess, in general, is not considered a widespread health concern [51]. HQ for Hg in the different population groups is very close to the reference value. However, the tendency of these metals to bioaccumulate in aquatic organisms could have future adverse effects in the ecosystem and also on the health of the surrounding populations [40,41,52]. Consequently, given that these values are above or below the reference values depending on the variable studied (i.e., EWI, MFW, or MeHg_PSL_), this shows that there is no evidence of a potential health risk from MeHg exposure in the population groups.

In general, the HQ results indicate that the WCA and the RP groups show a potential health risk from the intake of Cu and Hg through fish consumption, however for Hg the values of EWI, MFW, and MeHg_PSL_ suggest a low risk to human health. Copper is an essential element for the formation of hemoglobin and some enzymes in humans; however, high intake can damage the liver and kidneys [53]. That is why the chronic intake of small amounts of heavy metals can cause non-cancer risks, such as neurological problems, headaches, and liver and kidney diseases [54].

Furthermore, this study is the first report on arsenic As and its risk assessment in fish in Colombia, and knowing that about 90% of human exposure to As is due to the intake consumption of fish, shellfish and/or other marine organisms [55], it should be considered in future evaluations of environmental contaminants along with its speciation (organic and inorganic As), especially in different aquatic organisms of commercial interest. The reason is that since this metalloid bioaccumulates and biomagnifies through food chains, so the accumulation of As in tissues can cause chronic diseases and potential health damage to the population [56].

This result indicates that the species *Mugil incilis* of a detritivorous feeding habit could have a direct relationship between heavy metal accumulation and feeding habit, as there is no relationship pattern with the trophic level concerning the carnivorous species. As it is a species with high consumption (77%) based on the population survey, it can have significant adverse effects on human health due to the bioaccumulation of heavy metals. Therefore, the consumption of species with a lower MPI, such as *Eugerres plumieri*, is recommended. However, the rate of consumption of this species must be lower compared to what was indicated by the population survey evaluation (92%) since this species of a euryphagous food habit can become bioaccumulative with a higher proportion of heavy metals.

## 5. Conclusions

Concentrations of heavy metals in fish muscle in Ciénaga Grande de Santa Marta were low compared to those reported in other regions of the world and the maximum levels established by national and international monitoring organizations for fish consumption. Furthermore, these vary according to the species with no statistically significant difference and do not maintain a clear relationship with the trophic level and bioaccumulation. The hazard quotient for Cu exceeds the limit (HQ > 1) for the groups of women of childbearing age and the remaining population, indicating a potential risk for these two population groups from fish consumption. Lowering fish consumption and changing the diet would be the ideal recommendation. However, limiting fish consumption and promoting dietary replacement is not an option in most cases for populations that depend on this resource for most of their diet. Therefore, the corresponding environmental and health authorities are urged to take corrective action on behalf of these populations, which in most cases, have these fish species as their only source of protein. Besides, monitoring different aquatic organisms should be continued to establish relationships in the food chain and implement public education strategies to address fish consumption.

## Figures and Tables

**Table 1:** Concentrations of heavy metals (µg/kg) found in fish species assessed and values reported in other studies worldwide.

Scientific Name	N	Habit	Mean ± SD								MPI
			Total Length (cm)	Weight (g)	As	Cd	Hg	Pb	Zn *	Cu *	
This study											
*Cathorops mapale*	35	C	24.3 ± 5.6	117.8 ± 26.1	108.7 ± 44.8	8.7 ± 6.2	18.1 ± 14.6	38.4 ± 32.8	6.3 ± 1.2	8.7 ± 1.6	0.13
*Centropomus undecimalis*	26	C	33.6 ± 3.9	330.0 ± 120.3	114.6 ± 48.0	13.6 ± 18.3	28.2 ± 10.1	31.4 ± 36.8	5.9 ± 0.9	9.8 ± 1.3	0.15
*Elops smithi*	33	C	36.8 ± 2.2	284.4 ± 40.9	108.3 ± 56.7	7.9 ± 7.9	36.6 ± 44.0	49.3 ± 40.5	7.1 ± 2.9	18.1 ± 3.1	0.18
*Eugerres plumieri*	33	E	27.0 ± 4.9	158.0 ± 37.9	94.2 ± 57.9	11.0 ± 6.8	13.1 ± 11.3	33.8 ± 16.4	9.2 ± 4.0	7.1 ± 1.5	0.12
*Mugil incilis*	33	D	25.5 ± 3.5	113.0 ± 77.4	141.5 ± 109.3	20.8 ± 29.8	16.1 ± 21.4	36.3 ± 22.4	7.4 ± 2.1	9.4 ± 3.2	0.16
Species, country or site/Reference											
*Ariopsis bonillai*, CGSM, Colombia [29]		C	-	-	-	2000–4200	-	-	18–109	-	
*Mugil incilis*, CGSM, Colombia [7]		D	-	-	-	-	ND–51	-	-	-	
*Eugerres plumieri,* CGSM, Colombia [7]		E	-	-	-	-	ND–68	-	-	-	
*Mugil incilis*, Mallorquín swamp, Colombia [30]		D	-	-	-	60–160	-	-	16.6–27.8	0.41–0.94	
*Mugil incilis*, Mallorquín swamp, Colombia [31]		D	-	-	-	-	-	650–2030	13.8–21.3	0.09–0.8	
*Ariopsis felis*, Southern Gulf of Mexico [32]		C	-	-	-	-	-	10–250	-	-	
*Sardinella brasiliensis,* Rio de Janeiro, Brazil [33]			-	-	700–1200	6–40	-	60–900	6.7–12	1.1–4.7	
*P. bifasciatus*, San Pedrito Lagoon, Mexico [34]		D	96.2 ± 12.7	-	-	410	-		28.59	-	
*C. undecimalis,* Mallorquín swamp, Colombia [18]		C	24.6 ± 4.1	119.0 ± 46	-	30–130	100–170	70–290	11.1–22.6	0.16–1	
*E. plumieris,* Mallorquín swamp, Colombia [18]		E	16.6 ± 0.9	57.3 ± 9.9	-	70–160	140–290	80–110	3–4.7	0.48–2.02	
38 species of tropical marine fishes, Spratly Islands, China [35]			-	-	20,850	-	-	140	21.95	1.57	
*C. undecimalis,* Colombian Caribbean [36]		C	-	-	ND	ND	86	1472	-	-	
				FAO/WHO ^a^	-	50	500	200	40	-	
Permissible limit				EU ^b^	-	50–100	-	300	-	-	
				MHSP ^c^	-	100	-	300	-	-	

N: number of fish caught, MPI: metal pollution index, D: detritivorous, C: carnivorous, E: euryphagous, ND: no data. * µg/g; ^a^ [37], ^b^ [38], ^c^ [39]

**Table 2:** Estimates of the potential risk in the different age groups.

Heavy Metals	RfD ^a^	CHD		WCA		RP	
		EDI	CR_lim_	EDI	CR_lim_	EDI	CR_lim_
As	0.3	0.02	99.5	0.04	196.4	0.04	185.6
Cd	1	0.02	3359.9	0.05	6629.0	0.04	6265.8
Hg	0.1	0.04	189.8	0.09	374.5	0.08	353.9
Pb	4	0.06	4005.1	0.14	7901.9	0.13	7468.9
Zn	300	11.4	1582.6	27.4	3122.5	25.3	2951.4
Cu	40	16.8	153.8	40.5	303.4	37.4	286.7
Potential risk by consumption of fish with methylmercury							
		EWI (µg/kg/week)		MeHg_PSL_ (µg/g)		MFW (kg)	
CHD		0.25		0.130		3.04	
WCA		0.60		0.117		12.0	
RP		0.55		0.108		11.3	

CHD: children, WCA: women of childbearing age, RP: the remaining population, RfD: oral reference dose (μg/kg BW/day), EDI: estimated daily intake (µg/kg BW/day), CRlim: consumption rate (g/day), EWI: estimated weekly intake of MeHg (µg/kg BW/week), MeHg_PSL_: the permissible safety level for Hg (µg/g), and MFW (kg): the maximum amount of fish that can be consumed weekly per person. ^a^ Obtained from the Integrated Risk Information System, USEPA (2016), JECFA (http://apps.who.int/food-additives-contaminants-jecfa-database/Search.aspx), and ATSDR (http://www.atsdr.cdc.gov/substances/index.asp; http://www.atsdr.cdc.gov/HAC/PHA/reports/isladevieques_06272003pr/appendices1b.html).
